# Supplementary material for: Hot spot prediction in protein-protein interactions by an ensemble system
Source: BMC Syst Biol. 2018 Dec 31;12(Suppl 9):132. doi: 10.1186/s12918-018-0665-8 (PMC6311905; doi:10.1186/s12918-018-0665-8)
Supplement: Supplementary file 2 — All relevant MATLAB code. A simple Matlab implement of our predictor. (ZIP 19,742 kb) [file 12918_2018_665_MOESM2_ESM.zip › Additional file 2/Read ASA/ASA Property/SKEMPI/1KTZA.txt]

 NetSurfP - Protein Surface Accessibility and Secondary Structure Predictions

  

|  |  |  |
| --- | --- | --- |
|  |  | NetSurfP - Protein Surface Accessibility and Secondary Structure Predictions  Technical University of Denmark |

  


---

```
# For publication of results, please cite:
# A generic method for assignment of reliability scores applied to solvent accessibility predictions.
# Bent Petersen, Thomas Nordahl Petersen, Pernille Andersen, Morten Nielsen and Claus Lundegaard
# BMC Structural Biology 2009, 9:51 doi:10.1186/1472-6807-9-51
#
# Column 1: Class assignment - B for buried or E for Exposed - Threshold: 25% exposure, but not based on RSA
# Column 2: Amino acid
# Column 3: Sequence name
# Column 4: Amino acid number
# Column 5: Relative Surface Accessibility - RSA
# Column 6: Absolute Surface Accessibility
# Column 7: Z-fit score for RSA prediction
# Column 8: Probability for Alpha-Helix
# Column 9: Probability for Beta-strand
# Column 10: Probability for Coil
E E  1KTZA                  1    0.866 151.238   1.356   0.003   0.003   0.994
E N  1KTZA                  2    0.596  87.254   0.848   0.005   0.045   0.951
B C  1KTZA                  3    0.053   7.455  -2.318   0.019   0.141   0.840
E C  1KTZA                  4    0.375  52.678   0.957   0.022   0.552   0.426
E V  1KTZA                  5    0.401  61.572  -0.018   0.021   0.756   0.223
E R  1KTZA                  6    0.353  80.837   1.293   0.021   0.756   0.223
E P  1KTZA                  7    0.528  74.866   0.785   0.021   0.756   0.223
B L  1KTZA                  8    0.089  16.333   0.653   0.021   0.756   0.223
E Y  1KTZA                  9    0.347  74.175   0.811   0.018   0.846   0.136
B I  1KTZA                 10    0.046   8.436   0.692   0.021   0.756   0.223
E D  1KTZA                 11    0.295  42.510   0.495   0.087   0.683   0.230
B F  1KTZA                 12    0.045   8.991   0.775   0.231   0.330   0.439
E R  1KTZA                 13    0.394  90.226   1.138   0.307   0.165   0.527
E Q  1KTZA                 14    0.556  99.230   0.543   0.268   0.043   0.689
E D  1KTZA                 15    0.491  70.768  -0.445   0.354   0.048   0.598
B L  1KTZA                 16    0.191  34.990   0.314   0.184   0.043   0.773
E G  1KTZA                 17    0.405  31.905  -0.615   0.018   0.019   0.964
B W  1KTZA                 18    0.145  34.776   0.482   0.018   0.047   0.935
E K  1KTZA                 19    0.356  73.311   0.171   0.020   0.205   0.775
B W  1KTZA                 20    0.052  12.506   1.093   0.021   0.756   0.223
B V  1KTZA                 21    0.032   4.888   0.306   0.018   0.846   0.136
B H  1KTZA                 22    0.043   7.749   0.319   0.018   0.846   0.136
B E  1KTZA                 23    0.141  24.668   1.075   0.022   0.552   0.426
B P  1KTZA                 24    0.067   9.493  -0.094   0.004   0.197   0.799
E K  1KTZA                 25    0.587 120.705   0.439   0.018   0.047   0.935
E G  1KTZA                 26    0.473  37.249  -0.445   0.018   0.088   0.893
B Y  1KTZA                 27    0.149  31.777   0.427   0.020   0.205   0.775
E Y  1KTZA                 28    0.483 103.260   0.519   0.022   0.359   0.619
B A  1KTZA                 29    0.107  11.769  -0.613   0.021   0.451   0.528
B N  1KTZA                 30    0.235  34.345  -0.764   0.023   0.655   0.322
E F  1KTZA                 31    0.317  63.662   0.468   0.023   0.655   0.322
B C  1KTZA                 32    0.023   3.215  -0.362   0.023   0.655   0.322
E S  1KTZA                 33    0.364  42.637   0.055   0.022   0.552   0.426
B G  1KTZA                 34    0.087   6.863  -3.643   0.004   0.514   0.481
E P  1KTZA                 35    0.499  70.794   0.045   0.004   0.420   0.576
B C  1KTZA                 36    0.031   4.395  -0.212   0.021   0.279   0.699
E P  1KTZA                 37    0.529  75.023  -0.025   0.020   0.205   0.775
E Y  1KTZA                 38    0.311  66.375  -0.259   0.018   0.047   0.935
E L  1KTZA                 39    0.308  56.322  -0.254   0.018   0.019   0.964
E R  1KTZA                 40    0.480 109.989  -0.693   0.018   0.019   0.964
E S  1KTZA                 41    0.580  67.941  -2.028   0.018   0.019   0.964
E A  1KTZA                 42    0.694  76.446  -2.061   0.018   0.019   0.964
E S  1KTZA                 43    0.553  64.858  -0.999   0.018   0.019   0.964
B A  1KTZA                 44    0.257  28.288  -0.828   0.018   0.047   0.935
E S  1KTZA                 45    0.484  56.713   0.534   0.005   0.045   0.951
B P  1KTZA                 46    0.110  15.666  -1.646   0.004   0.085   0.910
B C  1KTZA                 47    0.078  10.965  -0.487   0.005   0.336   0.660
B C  1KTZA                 48    0.041   5.728  -0.801   0.022   0.552   0.426
B V  1KTZA                 49    0.180  27.697   1.079   0.022   0.552   0.426
B P  1KTZA                 50    0.104  14.786  -0.381   0.022   0.359   0.619
E Q  1KTZA                 51    0.346  61.778   0.246   0.022   0.359   0.619
E D  1KTZA                 52    0.510  73.549   0.557   0.022   0.359   0.619
B L  1KTZA                 53    0.222  40.667  -0.165   0.005   0.262   0.733
E E  1KTZA                 54    0.483  84.450  -0.175   0.004   0.085   0.910
E P  1KTZA                 55    0.398  56.434  -0.842   0.004   0.197   0.799
B L  1KTZA                 56    0.070  12.872   0.472   0.004   0.616   0.381
B T  1KTZA                 57    0.211  29.335   0.420   0.002   0.816   0.182
B I  1KTZA                 58    0.033   6.179   0.232   0.001   0.900   0.099
B L  1KTZA                 59    0.058  10.601   1.110   0.001   0.959   0.040
B Y  1KTZA                 60    0.079  16.840   0.633   0.000   0.983   0.017
B Y  1KTZA                 61    0.143  30.602   1.101   0.001   0.959   0.040
B V  1KTZA                 62    0.149  22.886   0.352   0.004   0.616   0.381
E G  1KTZA                 63    0.776  61.079   0.456   0.005   0.015   0.979
E R  1KTZA                 64    0.772 176.674  -0.001   0.003   0.003   0.994
E T  1KTZA                 65    0.480  66.631   0.238   0.004   0.197   0.799
E P  1KTZA                 66    0.318  45.181   0.862   0.003   0.718   0.279
E K  1KTZA                 67    0.280  57.534   1.629   0.011   0.918   0.071
B V  1KTZA                 68    0.193  29.695   0.634   0.006   0.962   0.032
E E  1KTZA                 69    0.312  54.506   1.605   0.001   0.900   0.099
E Q  1KTZA                 70    0.528  94.265   1.509   0.002   0.816   0.182
B L  1KTZA                 71    0.142  26.055   1.185   0.005   0.262   0.733
E S  1KTZA                 72    0.739  86.611   1.068   0.018   0.019   0.964
E N  1KTZA                 73    0.686 100.357   0.598   0.018   0.019   0.964
B M  1KTZA                 74    0.093  18.529   0.700   0.022   0.359   0.619
E V  1KTZA                 75    0.309  47.447   1.741   0.018   0.846   0.136
B V  1KTZA                 76    0.114  17.522  -0.018   0.011   0.918   0.071
E K  1KTZA                 77    0.540 111.140   1.082   0.018   0.846   0.136
E S  1KTZA                 78    0.562  65.890   0.669   0.023   0.655   0.322
B C  1KTZA                 79    0.101  14.124  -0.647   0.021   0.451   0.528
E K  1KTZA                 80    0.464  95.445  -0.541   0.005   0.262   0.733
B C  1KTZA                 81    0.210  29.512  -1.340   0.004   0.085   0.910
E S  1KTZA                 82    0.781  91.580  -0.657   0.003   0.003   0.994

Explain the output.  Go back.
```

---
